# Updated Dietetic Prescribing Guidelines When Naso‐Gastric Tube Feeding Under Physical Restraint Is Required: A Modified Nominal Group Technique Study

**DOI:** 10.1111/jhn.70242

**Published:** 2026-04-09

**Authors:** Sarah J. Fuller, Ursula Philpot, Helen West

**Affiliations:** ^1^ Northamptonshire Healthcare NHS Foundation Trust Northampton England; ^2^ Imperial College London London England; ^3^ Leeds Beckett University Leeds England; ^4^ Faculty of Nursing Midwifery and Palliative Care King's College London London England

**Keywords:** mental health, naso‐gastric tube feeding, nominal group technique, physical restraint, prescribing, restrictive practices

## Abstract

**Introduction:**

Naso‐gastric tube (NGT) feeding under physical restraint is a lifesaving clinical intervention that is sometimes needed in mental health patients, particularly those with restrictive eating disorders. Initial guidance was published in 2020 and subsequently reviewed and updated to support dietitians in this specialist area.

**Methods:**

Using purposeful recruitment, we brought together specialist mental health dietitians from England, Wales, Scotland and Northern Ireland who work in inpatient child and adolescent mental health services or adult inpatient mental health services. We followed modified nominal group technique in the update of these guidelines. Nominal group technique has four stages. Stage one is for silent idea generation, stage two facilitates round‐robin discussion, stage three is the discussion and refining of ideas and stage four the voting and ranking of ideas to generate consensus.

**Results:**

We have updated the clinical guidelines for dietetic prescribing when NGT feeding under physical restraint for a mental health condition is needed. This updated guideline promotes the least restrictive practice and encourages dietetic prescribing to be proportionate to medical risk.

**Conclusion:**

We have provided an updated version of the dietetic prescribing guidelines when NGT feeding under physical restraint is needed. These guidelines now apply to mental health patients in general, not just those with Anorexia Nervosa and incorporate expert clinical opinion regarding strategies to wean off this clinical intervention.

## Summary

- The dietetic prescribing guidelines are applicable to other restrictive eating disorders and mental health diagnoses when severe malnutrition may be seen alongside resistance to appropriate physical health restoration.
- Teams should ensure they are actively engaging in weaning strategies when NGT feeding past the point of medical stabilisation.
- Enteral feeding policies should be updated in both mental health and acute ward settings to include NGT feeding under physical restraint and the change in dietetic prescribing practices that is needed.

## 1 Introduction

In 2020, the first dietetic guidelines regarding naso‐gastric tube (NGT) feeding under physical restraint for those with Anorexia Nervosa (AN) were published [1]. The guidelines suggested changes to dietetic prescribing to shorten time spent in physical restraint and to ensure that dietetic prescribing met legal principles. Since then, understanding of the patient population, feeding practices, and environments that NGT feeding under physical restraint occurs in England has developed.

Research conducted in mental health wards in England identified 622 patients receiving this clinical intervention in a 1‐year period (2020–2021) [2]. Another notable finding was the range of mental health diagnosis and co‐morbidities where NGT feeding under physical restraint was used [2]. In addition to AN, patients with other eating disorders such as bulimia nervosa, other specified feeding or eating disorder and disordered eating as well as other psychiatric conditions, for example, obsessive‐compulsive disorder, pervasive arousal withdrawal syndrome, psychosis also received this invention. Importantly, this clinical intervention was used past the point of medical stabilisation, with the mean duration being 29.1 weeks [2]. Similar findings were reported in English paediatric wards, where 144 young people received NGT feeding under physical restraint between 2022 and 2023 [3]. Again, with a similar range of mental health diagnoses, and past the point of medical stabilisation, with the mean duration of 60.2 days [3].

Research suggests that those who need NGT feeding under physical restraint are more likely to have a persistent eating disorder diagnosis at 5‐year follow up [4]. Research also indicates that the emotional and psychological impact of NGT feeding under physical restraint extends beyond the patients themselves. Studies seeking to understand the impact on patients, their parents/carers and clinical staff ‐ including uniformed security staff—suggest all involved may experience significant distress and potential trauma because of this clinical intervention [5, 6, 7, 8]. Furthermore, it has been reported that a significant number of people with ED's and other mental health conditions also have co‐occurring neurodivergence [9, 10]. Evidence suggests that Neurodivergent individuals often have poorer treatment outcomes and experience eating disorder symptoms over a longer period when compared with the neurotypical eating disorder population [9, 11, 12]. The reasons for this are poorly understood. However, sensory processing, communication differences, and preference for sameness, routine, or predictability may put neurodivergent individuals at increased risk of harm when using NGT feeding under restraint. Therefore, considering individual needs is essential for minimising iatrogenic harm.

Legal frameworks across the United Kingdom (UK) emphasise acting in the patient's best interest, using least restrictive practice, using reasonable force and interventions being proportional to risks. However, evidence that NGT feeding under physical restraint often continues beyond medical stabilisation [2, 3, 13], and may cause psychological harm, created challenges for multi‐disciplinary teams (MDT's). Updating the existing dietetic guidelines is therefore necessary to support best clinical practice, including safe strategies for reducing and discontinuing this clinical intervention.

## 2 Methods

### 2.1 Study Design and Participants

We developed a project group of expert dietitians with experience of prescribing NGT feeds for those who needed physical restraint to maintain their safety during the feed. These dietitians were purposefully recruited to reflect a range of mental health inpatient environments that included children's units, general adolescent units, adolescent eating disorders units, and adult eating disorder units or adult psychiatric acute admissions wards. Participating dietitians represented practice within England, Wales, Scotland and Northern Ireland for the National Health Service or independent sector mental health wards.

### 2.2 Materials and Procedure

We used nominal group technique as this is a well‐established, simple and intuitive process widely used for group decision‐making within healthcare [14]. We facilitated a structured meeting of clinical experts and followed the nominal group technique as outlined in Box 1. Step one was modified by sharing the three research questions prior to the meeting, so all participants could brainstorm for a longer period. Step two and three occurred as defined. Step four was modified as any disagreements were resolved through discussion and consensus. Voting was not required as the group did not produce multiple options for practice changes and agreed regarding the suggested solutions.

Box 1 **The four step process of nominal group technique.**

1

**Table:** 

1. Silent idea generation: team members brainstorm independently for 10 min without discussion. 2. Round‐robin discussion: Each participant shares one idea at a time in a structured interruption‐free format. 3. Discuss and refine Ideas: The group clarifies and improves each idea while combining similar ones. 4. Vote and ranking: using a point‐based system to rank the ideas and decide the best solution

## 3 Results

We recommend the application of these dietetic prescribing guidelines to include other restrictive eating disorders and mental health diagnoses when severe malnutrition may be seen alongside resistance to appropriate physical health restoration.

### 3.1 Prevention of NGT Feeding Under Restraint

Oral nutrition should be offered initially via structured but flexible meal plans, and these will be sufficient for most patients to establish an appropriate oral intake. However, some will need bespoke plans that account for an individual's sensory specificities, cultural requirements and historic preferences [15]. Consider using foods/snacks/drinks from home if they are not available via hospital catering and will be accepted with less distress. It is important that meal plans are complemented with specialist meal support to maximise oral intake and promote medical stability.

At this stage of treatment, the variety within a meal plan is less important than the total overall energy prescription, especially in life‐threatening situations. Variety of diet and macronutrient balance can be discussed and worked on later. Dietitians may be concerned regarding colluding with an eating disorder when supporting highly bespoke meal plans. However, if reasonable adjustments are requested and agreed between the patient and the MDT that they will aid establishing a safe oral diet then they should be tried. It will soon become clear if any request to alter a meal plan is facilitating dietary avoidance and is in fact unhelpful. Dietitians should be alert to persistent attempts to trade down the overall nutritional value of any meal or snack and ensure that any changes to meal plans are of the same overall caloric value.

Whilst an oral diet is always the preferred option, oral nutritional supplements (ONS) can be helpful in establishing safe oral intake and should be offered as replacements or alternatives to equivalent energy of the meal plan. It is rarely helpful, unless specifically agreed with the patient, to have meal plans that involve additional energy prescribed for non‐completion of meal plans, that is, a snack is 200 kcal and the replacement is 300 kcal, as this is often perceived as a punishment for not being able to eat enough or being distressed.

Many patients will accept NGT feeding without any physical restraint to maintain their safety [16, 17]. Clinical experience suggests that some patients report that NGT feeding is a relief for them as they do not have to experience the emotional distress from eating. However, for a minority, their physical health continues to be compromised, and they resist all opportunities to establish a safe nutritional intake and NGT feeding under physical restraint may be required.

### 3.2 Nasogastric Tube Feeding Under Restraint

Prior to any restraint for NGT feeding, the patient should be offered specialist meal support to take food, nutritional supplements and or water orally. This ensures that least restrictive practice is being carried out and that every opportunity to take oral food and fluid has been offered. For some, explaining that a NGT feed under physical restraint is being planned for a specific time may be enough for them to establish a safe oral intake.

Given the high risk and complexity of patient care and decision making when NGT feeding under restraint is being considered. If this is outside your usual scope of practice, seek supervision from more experienced dietitians.

### 3.3 NG Feeding Tube

Selecting a wider NGT such as 12‐14Fr can ease delivery of the NGT and support quicker delivery of push syringe boluses. Smaller size NGT's can be used when patients experience discomfort.

It is not routine practice to use NGT with nasal bridals [18], due to the risk of them being pulled out and the septum being torn as a result.

If the NGT remains in situ, be aware it should not be left for longer than indicated by manufacturing guidelines because of the risk of the NGT deteriorating and nasal pressure sores.

### 3.4 Feeding Modality

The feed should be delivered via a push syringe bolus and not gravity bolus or enteral pump to ensure delivery in the shortest timeframe, minimising the time spent in physical restraints. In very rare circumstances, there may be a medical reason that pushes syringe boluses are not tolerated, and enteral pump feeds may need to be considered. For some patients, they may only need low‐level restraints such as hand holds to maintain their safety and enteral pump feeding may be appropriate. However, for those who need higher levels of physical restraints, this may not be appropriate given the length of time taken to deliver the feed.

### 3.5 Feeding Rate

In specialist mental health in‐patient services, common practice suggests that it is safe to start at one 50‐mL syringe per minute and increase to two 50‐mL syringes per minute as tolerated. Typically, 1000 mL boluses can therefore be safely delivered within 10 min.

Bolus feeds can be started at 500 mL per bolus; this can be increased every day by 100–200 mL up to 1000 mL as required. However, when working with children it would be appropriate to adopt a more cautious approach with the initial bolus size and rate of increase. Delivering boluses over 1000 mL has been managed safely but it is not viewed as standard practice and should only be considered if the patient is tolerating 1000 mL with no concerns.

### 3.6 Feed Prescription

The use of a high calorie small volume ONS (2–2.4 kcal/mL) ensures maximum nutrition can be delivered in the smallest volume and time. High calorie small volume enteral feeds (2 kcal/mL) can be a helpful alternative if the viscosity of the feed is an issue.

Modular supplements can be considered to aid a reduction in total feed volume. These can be either via a syringe bolus themselves or added to the ONS/enteral feed as a mixture.

Fat based supplements (4.5–5 kcal/mL) are often used, especially to promote optimal energy intake in smaller volumes. Start at 30–50 mL per feed and increase as tolerated. In practice volumes up to 200 mL have been tolerated in some patients. Furthermore, research suggests that hospitalised malnourished young people (up to age 25 y) tolerated a lower carbohydrate/high fat formula (28% carbohydrate, 56% fat) and this protected from hypophosphatemia in the first week compared to when using a standard enteral formula [19].

For those who do not tolerate the addition of fat‐based supplements, consider adding in carbohydrate‐based supplements (3.8 kcal/mL) to reduce the total volume of feed as these tend to have less side effects. Caution should be used when using these carbohydrate supplements in adult patients at high risk of refeeding [20].

### 3.7 Fluid Prescription

For some who require this clinician intervention, they may have restricted both their nutrition and hydration. Separate bolus feeds for hydration may need to be considered and can be psychologically less distressing and not require physical restraints. Electrolyte solutions can be added to water boluses to promote rehydration as needed. In cases when fluid is being refused, a lower threshold for intervention may be required due to increasing the risk of medical instability.

Water can also be added to the feed bolus to reduce the viscosity of feed thus aiding the administration of the feed via 50 mL syringe.

### 3.8 Feeding Pattern

Consensus was that the number of feeds a day is usually reduced to two, reducing episodes of distress and risk to the patient as well as staff. However, one feed a day can be considered in circumstances when there are no concerns around the patient's physical health and they are maintaining their hydration orally or via the NGT without physical restraint. Areas for concern regarding this feeding pattern would include patients who are extremely low weight, those who are symptomatic with persistent low blood sugars and at risk of refeeding syndrome [18]. Some degree of clinical judgement is required to assess those individuals where additional caution is required.

Clinical experience suggests that once the feeding pattern has been decided, that is, one or two feeds a day, patients benefit from knowing when their NGT feed is being planned for. Working with patients to identify the best time of day for them can also be helpful. Some may prefer it in the morning, reducing their anticipatory anxiety of ‘when will this happen’, whereas others may prefer it towards the end of the day to give themselves more opportunities for a safe oral intake.

### 3.9 Monitoring and Review

#### 3.9.1 Feed Tolerance

A patient's tolerance to syringe bolus feeding regimen should be monitored daily and adjusted if a patient has any adverse symptoms (e.g., stomach aches, diarrhoea, vomiting) related to bolus volume or rate.

For some, gently warming the feed by standing in warmed water, akin to warming infant formula bottles on a paediatric ward and as per manufacturers guidelines, can aid both the viscosity of the feed and reduce sensory issues if feeds are traditionally stored in cool environments.

### 3.10 Biochemical Monitoring

If a patient is on one or two feeds a day for a prolonged period it would be advisable to consider biochemical monitoring for any long‐term adverse effects of receiving nutrition in this way.

### 3.11 Refeeding Syndrome

New guidelines regarding management of refeeding syndrome in those with eating disorders were published in 2022 [4]. This ‘medical emergencies in eating disorders’ guidance advises that patients at risk of refeeding syndrome should be medically monitored alongside being given the appropriate prophylactic vitamin and mineral supplementation.

For patients under the age of 18‐years, guidance now suggests starting at 1400 kcal/day, increasing by 200 kcal/day until at least 2400 kcal/day is safe [4]. Whereas for adult patients, those at the highest risk of refeeding syndrome should start at 10–20 kcal/kg/day, increasing by 5 kcal/kg/day every 2 days until 60 kcal/kg/day and those at low or medium risk should start at 30‐35 kcal/kg/day and increase in the same way [4]. These refeeding protocols can be used when NGT feeding under physical restraint is required. Again, in the adult population, caution regarding additional carbohydrate should be used for those at high risk of refeeding syndrome.

### 3.12 The Importance of MDT Working

No single clinician within an MDT can safely manage the combined medical, nutritional, refeeding, and mental health risks involved. Therefore, a loan clinician should not decide to start NGT feed under physical restraint outside of a single lifesaving feed. Ideally this intervention should follow a consultant‐led MDT discussion which weighs risks and benefits for the patient alongside consultation with the patient and their family, ensuring this is both lifesaving and proportionate.

Clear documentation of the decision‐making process, clinical rationale, with a full physical health and mental health risk assessment (including the risks of in adequate nutrition) is essential. Legal clarification may be required if there is a disagreement.

Patients receiving NGT feeding under physical restraint should have this decision reviewed daily by the MDT ‐ is this needed today? Teams should use a formulation‐based approach: first, consider the function of the food refusal; second, set clear clinical goals for using NGT feeding under restraint; and third, develop care plans that support returning to either accept NGT feeding or return to an oral intake. Treatment goals should be developed collaboratively with the patient and their family and be communicated clearly in line with the patient's communication needs and preferences. These should also include agreed criteria for when NGT feeding under physical restraint should be stopped.

### 3.13 Restraint Safety and Distress

If a restraint for a NGT feed has lasted longer than 20 min it would be reasonable for the MDT to discuss if a medical review is needed. Furthermore, the MDT can consider a medication review to see if any pharmacological adjuncts could be prescribed to reduce distress.

### 3.14 Nutritional Adequacy

Most ONS and enteral feeds are nutritionally complete in specific volumes. However, if NGT feeding under physical restraint for prolonged periods, these may not be met and therefore nutritional analysis should be conducted to identify if any additional vitamins and minerals are required. Prescribable liquid vitamin and mineral supplements can be given via the NGT as needed to ensure nutritional adequacy. Likewise, consider a ONS/enteral feed with added fibre if this clinical intervention is being used for a prolonged period.

### 3.15 Policy Development

All physical and mental health services should include in their enteral feeding, nutrition, and hydration policies guidelines for treating patients who require NGT feeding under physical restraint.

### 3.16 Considerations When NGT Feeding Under Physical Restraint Past the Point of Medical Stability

Research suggests that some patients are NGT fed under physical restraint past the point of medical stabilisation and initiating full health restoration. In line with legal principles of least restrictive practice and proportionality to risk, dietitians can change their prescribing of NGT feeds once a patient is medically stable and there is no longer a threat to life. The following strategies (Box 2) can be given consideration.

Box 2 **Weaning strategies.**

1

**Table:** 

1. Temporarily stopping NGT feeding under physical restraint with close monitoring of the patient's physical health observations to see if meal support can establish a safe minimal oral intake (oral diet or ONS) during this time. 2. Giving 7‐days of nutrition across 6‐days to allow a day where they are not receiving NGT feeding under physical restraint to reduce distress, enhance opportunities to engage in therapeutic opportunities and where safe to do so leave off the unit. 3. Temporarily switch to a weight maintenance prescription, to ensure medical stability, but not facilitate significant amounts of weight and gradually establishing an oral diet or ONS. 4. Consider continuing to facilitate some weight restoration but pause at a safe rather than optimal weight, to work on establishing oral diet or ONS. 5. Overnight feeding may be considered to reduce distress and mitigate the need for restraint for NGT feeding.

## 4 Discussion

This update to the dietetic guidelines regarding NGT feeding under physical restraint represents further improvement in practice when this clinical intervention is needed. In broadening the scope of these guidelines to wider mental health diagnoses, not just AN, as we recognise that physical health compromise can occur across multiple mental health diagnoses.

The original guidelines used a modified Delphi method [1], which supports the development of consensus but is time‐consuming. Therefore, nominal group technique was chosen, enabling agreement in a single session and allowing a balance of independent thinking and for group interaction and discussion [14].

Whilst NGT feeding under physical restraint can be lifesaving, research suggests that it is also used past the point of medical stabilisation for some. This needs to be balanced with the risk of iatrogenic harm and the justification for continuing becomes legally tenuous [21]. This update now includes weaning strategies for MDTs to consider that meet overarching legal principles within UK law. These guidelines advocate for the continued reassessment of medical and psychiatric risks regarding the need for continuing this highly restrictive clinical intervention, thus ensuring proportionality to the risks presented. It is hoped that these strategies, alongside formulation led MDT discussions, will encourage positive risk taking to enable patients to re‐establish a safe oral intake.

Regardless of NGT feeding pattern, patients require meaningful activities and therapeutic opportunities during their day to promote their overall mental wellbeing. Additionally, there are potential therapeutic opportunities that can be facilitated when either stopping or having a day off from a NGT feed under physical restraint. Both strategies should allow a patient to move out of a ‘out of control’ state (from being NGT fed under physical restraint) and towards their zone of regulation [22]. In turn, this can generate more opportunities to facilitate effective meal support, or engage in relaxation strategies, or time to engage in more therapeutic conversations around managing things differently. If medically and psychiatrically safe to do so, time off the ward can be facilitated. This would allow someone to see life outside the unit and their illness, or time to rebuild relationships with family and friends as these often suffer during treatment. These experiences may strengthen motivation, connection, recovery or enable someone to manage their NGT feed differently.

Likewise, persisting with prolonged weight restoration may be psychologically too difficult for some to also restart eating or drinking ONS. Therefore, reducing the feed prescription either quickly after medical stabilisation and some weight gain or at a mutually agreed sub‐optimal point can allow some patients to move off this clinical intervention and return to oral diet or ONS. With both strategies further weight restoration can then be considered once the patient is in a psychologically safer state.

With all these strategies, the working group agreed that patients need to be monitored daily to ensure any change in feeding strategy is providing a meaningful therapeutic benefit and is not causing further deterioration or worsening of their physical and or mental health.

Clinical experience suggests that the patient group who are NGT fed under physical restraint for a psychiatric illness, typically do not present with the traditional medical risks when overnight NGT feeding is considered. Enhanced supportive observations such as 1:1 monitoring may be less restrictive and proportionate to the potential risks from physical restraints. Furthermore, the patient may experience bolus feeding as unhelpful from a sensory perspective, exacerbating their distress, and therefore enteral pump feeding may facilitate the patient managing their feed without the need for physical restraints.

The working group notes that both the treating team and patients can become rigid in their thinking regarding NGT feeding under physical restraint. The treating team may feel the need to continue with this clinical intervention as their patient has not established a safe oral intake. Conversely, the patient may struggle to achieve this as they continue to receive NGT feeds under physical restraint. The treating team may be well placed to take positive risks which in turn may allow their patient to do something different. Furthermore, it is important for the MDT to understand the function of their patient's food refusal, and possibly their need for physical restraints, as malnutrition is rarely the only unmet need leading to NGT feeding under physical restraint and meeting other functional needs the patient may have is essential in reducing reliance on this clinical intervention.

### 4.1 Strengths and Limitations

Within the working group there was a broad range of dietitians from different countries, across age specific services and NHS and private sector. Therefore, this is likely to be an adequate representation of current specialist dietetic practice within the UK. Furthermore, this updated dietetic guideline continues to move dietetic prescribing practice to be in line with legal principles of care that apply to these patients. However, this is practice‐based evidence due to the lack of evidence base in this clinical area in which to base clinical practice decisions. Furthermore, this guideline update only focuses on dietetic prescribing and to date there are no MDT wide guidelines regarding this clinical intervention.

### 4.2 Further Research

Further research is needed to evaluate the effectiveness of these updated guidelines, especially the weaning strategies, and identification of predictors of successful transition to oral intake will be important to further refine best dietetic practice.

#### 4.2.1 Summary and Key Recommendations

If a patient requires NGT feeding under physical restraint secondary to a mental health diagnosis, dietitians should amend their traditional prescribing practices to meet the legal requirements of least restrictive practice and proportionality to risk. Therefore, we recommend:

1. These guidelines apply to mental health conditions where restrictive eating has led to severe malnutrition, not just AN.
2. It is important to prioritise oral nutrition using structured but flexible meal plans alongside specialist meal support and NGT feeding under physical restraint should only be considered in situations of imminent medical risks where all other forms of support and nutrition have been exhausted, ensuring least restrictive practice.
3. High calorie small volume oral ONS (2–2.4 kcal/mL) and modular supplements can be used in combination to minimise feed volumes and ensure that delivery by push syringe bolus is as quick as possible.
4. Start with cautious bolus volumes, for example, 500 mL and build up as tolerated up to 1000 mL.
5. In medically stable patients, consider weaning strategies to avoid continuation of this clinical intervention that is no longer proportionate to risks.

## Author Contributions

S.F., U.P., and H.W. conceived the study idea and carried out the research process. All three main authors (S.F., U.P., and H.W.) discussed the results, wrote the manuscript together, and contributed to the final version of the manuscript.

## Funding

The authors have nothing to report.

## Working Group

**England**

Hannah Barlow—Highly Specialist Paediatric Dietitian, Manchester University NHS Foundation Trust, England.

Sarah Elder—Advanced Practice Dietitian in Adult Eating Disorders, Tees Esk Wear Valleys NHS Foundation Trust, England.

Nicole Deklerk—Specialist Eating Disorders Dietitian, North London NHS Foundation Trust, England.

Lucy Gardner—Professional Lead Dietitian for mental health, Oxford Health NHS Foundation Trust, England.

Jayne Hilton—Clinical Specialist Dietitian, Nottinghamshire Healthcare Foundation Trust, England.

Marian Galvez Cazorla—Specialist Dietitian for CAMHS in‐patients, Northamptonshire Healthcare NHS Foundation Trust, England.

**Wales**

Lisa Simon—Highly Specialist CAMHS Dietitian, Cwm Taf Morgannwg University Health Board, Wales.

Claire McCluskey—Specialist Adult Eating Disorders Dietitian, Elysium Healthcare, Wales.

**Scotland**

Susan McDonald—Specialist Dietitian, Greater Glasgow and Clyde NHS Trust, Scotland.

Iain Scott—CAMHS Specialist Dietitian, NHS Lothian, Scotland.

**Northern Ireland**

Lorraine Craig—Clinical Specialist Dietitian, Belfast Health and Social Care Trust, Northern Ireland.

Lisa Ferris—CAMHS Mental Health Specialist Dietitian, Belfast Health and Social Care Trust, Northern Ireland.

## Ethics Statement

The authors have nothing to report.

## Consent

All participating dietitians provided informed consent to take part in the research process and to the publication of the results.

## Conflicts of Interest

The authors declare no conflicts of interest.

## Transparency Declaration

The authors affirm that this manuscript is an honest, accurate and transparent account of the guidelines being reported. No important aspects have been omitted. No funding was received for the development of these guidelines or manuscript.

## Data Availability

The authors have nothing to report.
